# Reabilitação Cardíaca Baseada em Exercícios (RCBE): Novas Fronteiras no Pós-Novo Coronavírus

**DOI:** 10.36660/abc.20230120

**Published:** 2023-03-22

**Affiliations:** 1 Hospital de Clínicas de Porto Alegre Grupo de pesquisa em cardiologia do exercício Porto Alegre RS Brasil Hospital de Clínicas de Porto Alegre – Grupo de pesquisa em cardiologia do exercício (CardioEx), Porto Alegre, RS – Brasil; 2 Hospital de Clínicas de Porto Alegre Grupo de pesquisa em cirurgia vascular e exercício Porto Alegre RS Brasil Hospital de Clínicas de Porto Alegre – Grupo de pesquisa em cirurgia vascular e exercício (VascoEx), Porto Alegre, RS – Brasil; 3 Hospital de Clínicas de Porto Alegre Serviço de fisiatria e reabilitação Porto Alegre RS Brasil Hospital de Clínicas de Porto Alegre – Serviço de fisiatria e reabilitação, Porto Alegre, RS – Brasil; 4 Universidade Federal do Rio Grande do Sul Departamento de cirurgia Porto Alegre RS Brasil Universidade Federal do Rio Grande do Sul, Departamento de cirurgia, Porto Alegre, RS – Brasil

**Keywords:** COVID-19/complicações, Coronavirus, Pandemia, Reabilitação Cardíaca, Recuperação da Função Fisiológica, Atividade Física, Terapia por Exercício

A pandemia da doença de coronavírus 2019 (COVID-19), além de repercussões físicas, emocionais e sociais gerou importantes alterações nas medidas não farmacológicas de auxílio ao tratamento de pacientes com doenças cardíacas, como é o caso da Reabilitação Cardíaca Baseada em Exercícios (RCBE). A incapacidade associada à síndrome pós-COVID-19 parece ter um impacto considerável nos serviços de saúde, pois o tratamento desses pacientes implica uma demanda muito grande de cuidados e um alto custo econômico.^1^

Evidências científicas demostraram uma associação estreita do comportamento sedentário com desenvolvimento e agravo de doenças cardiovasculares (DCV), além de mortalidade precoce. Muitas dessas mortes poderiam ser evitadas ou postergadas com cuidados preventivos e medidas terapêuticas, onde a RCBE é um pilar fundamental.^2^^,^^3^

Os serviços de RCBE foram temporariamente suspensos pela política crescente de distanciamento social que levou a vários bloqueios sucessivos, tentando prevenir a propagação da infecção. Um novo cenário se estabeleceu pela impossibilidade de os pacientes irem de forma presencial aos centros de tratamento, o que já era limitado por vários fatores, incluindo geografia, tempo, finanças e número de centros de reabilitação disponíveis no Brasil. Outras oportunidades sociais para pacientes com DCV praticarem exercícios com quantidade e intensidade recomendadas de atividade física regular também foram interrompidas.^4^ O COVID-19 apresentou um risco duplo para esses indivíduos. Em primeiro lugar, comorbidades cardiovasculares pré-existentes expondo pacientes a alto risco de desfechos adversos em caso de infecção pelo coronavírus. Em segundo lugar, o combate aos fatores de risco modificáveis que podem ser controlados com atividade física regular também ficaram prejudicados.^5^

Os atendimentos em RCBE requerem transformações que reinventem o modelo de relacionamento paciente-terapeuta, a fim de atender com qualidade as necessidades das pessoas, fazendo com que retornem ao nível da melhor capacidade funcional e independência possível da maneira mais eficaz, eficiente e segura, buscando reduzir as consequências diretas e indiretas do COVID-19.^6^ Essas transformações, no entanto, não são uma tarefa fácil.

O estudo de Ghisi et al.,^7^ publicado nos ABC Cardiol, apesar de ter uma amostra pequena e utilizado um questionário online desenvolvido pelos autores, mostrou como as ameaças econômicas experimentadas pelos profissionais de saúde e seus programas, e a incapacidade de muitos pacientes de ter acesso e poder navegar no mundo virtual afetou sobremaneira os participantes da RCBE. Os autores avaliaram participantes de programas de RCBE sobre alfabetização em saúde, uso de tecnologia e acesso à internet e como eles perceberam sua saúde durante a pandemia, dentre outras coisas, 26% relataram agravo em sua condição cardíaca e sintomas como dor no peito, falta de ar, palpitações cardíacas, ansiedade e depressão. Indo além, também alertaram para o fato de que as instituições e os profissionais de RCBE devem trabalhar para desenvolver uma melhor abordagem prática para monitorar os pacientes cardiopatas virtualmente e personalizar os cuidados de prevenção, ajudando esses indivíduos em sua recuperação e na prevenção de eventos.^7^ Haskiah et al. também encontraram redução na capacidade de exercício e aumento no percentual de gordura em participantes que suspenderam seus tratamentos durante a pandemia de COVID-19. Sugerem que a reabilitação cardíaca remota possa ser uma alternativa eficaz à reabilitação cardíaca ambulatorial em períodos como a pandemia de COVID-19 e mesmo para pacientes que optem por essa abordagem.^8^ Apesar do encaminhamento ser recomendação Classe I, apenas entre 30-50% dos pacientes elegíveis são encaminhados para RCBE por seu cardiologista; e menos ainda, conseguem concluir um programa.^9^^,^^10^ Nakayama et al. identificaram no Japão, o que ocorre também no Brasil: o principal impedimento para frequentar programas de RCBE é a distância de casa para o local do programa e escassez de serviços estruturados.^10^^,^^11^

Um fator positivo deste novo cenário da RCBE supervisionada à distância é a implementação da chamada fase 4 de reabilitação com exercícios. Esta fase caracteriza-se pelo paciente não mais estando vinculado aos centros de tratamento de forma presencial e sim remota. Ownbey et al.,^12^ mostraram que não há diferenças significativas nos resultados dos pacientes, entre híbridos e presenciais, e indicam que a RCBE remota é uma adição viável ao atendimento presencial.^12^ No entanto, no Brasil, assim como em outros países,^13^ a maioria dos profissionais de saúde tem pouca ou nenhuma experiência na implementação de RCBE domiciliar e remota, além disso os convênios médicos e o próprio sistema único de saúde (SUS) normalmente não cobrem essa modalidade de atendimento. São necessários maiores investimentos em treinamento de profissionais e implementação de formas de monitoramento de exercícios à distância, educação para uma vida saudável e modificação de comportamento para melhorar a saúde geral.

A RCBE encontra-se numa fronteira, necessitando novas pesquisas na área, maiores investimentos que permitam manter um maior número de pacientes em atendimento tanto presencial quanto remoto, onde o maior impedimento se encontra no investimento financeiro, educação do paciente para sua automonitorização, permitindo assim uma maior segurança em relação à prescrição do exercício físico à distância.
