# Supplementary material for: Reabilitação Cardíaca Baseada em Exercícios (RCBE): Novas Fronteiras no Pós-Novo Coronavírus
Source: Arq Bras Cardiol. 2023 Mar 22;120(3):e20230120. [Article in Portuguese] doi: 10.36660/abc.20230120 (PMC10392857; doi:10.36660/abc.20230120)
Supplement: Translation

# Translation: Exercise-Based Cardiac Rehabilitation (ECBR): New Frontiers in the Post-Novel Coronavirus

**Affiliations:** 1 Porto Alegre RS Brazil Hospital de Clínicas de Porto Alegre – Grupo de pesquisa em cardiologia do exercício (CardioEx), Porto Alegre, RS – Brazil; 2 Porto Alegre RS Brazil Hospital de Clínicas de Porto Alegre – Grupo de pesquisa em cirurgia vascular e exercício (VascoEx), Porto Alegre, RS – Brazil; 3 Porto Alegre RS Brazil Hospital de Clínicas de Porto Alegre – Serviço de fisiatria e reabilitação, Porto Alegre, RS – Brazil; 4 Porto Alegre RS Brazil Universidade Federal do Rio Grande do Sul, Departamento de cirurgia, Porto Alegre, RS – Brazil

**Keywords:** COVID-19/complications, Coronavirus, Pandemics, Cardiac Rehabilitation, Recovery of Function, Physical Activity, Exercise Therapy

The coronavirus disease 2019 (COVID-19) pandemic, in addition to physical, emotional, and social repercussions, has generated important changes in non-pharmacological measures to aid in treating patients with heart disease, such as Exercise-Based Cardiac Rehabilitation (ECBR). The disability associated with the post-COVID-19 syndrome seems to have a considerable impact on health services, as the treatment of these patients implies a very high demand for care and a high economic cost.^1^

Scientific evidence has shown a close association between sedentary behavior and the development and worsening of cardiovascular disease (CVD) and early mortality. Many of these deaths could be avoided or postponed with preventive care and therapeutic measures, where EBCR is a fundamental pillar.^2,3^

EBCR services were temporarily suspended by the increasing policy of social distancing, which led to several successive lockdowns, trying to prevent the spread of the infection. A new scenario was established by the impossibility of patients going to the treatment centers in person, which was already limited by several factors, including geography, time, finances, and the number of rehabilitation centers available in Brazil. Other social opportunities for CVD patients to exercise at the recommended amount and intensity of regular physical activity were also cut off.^4^ COVID-19 presented a dual risk for these individuals. First, pre-existing cardiovascular comorbidities expose patients to a high risk of adverse outcomes in case of coronavirus infection. Second, the fight against modifiable risk factors that can be controlled with regular physical activity was also impaired.^5^

EBCR care requires transformations that reinvent the patient-therapist relationship model in order to meet people's needs with quality, making them return to the level of the best functional capacity and independence possible in the most effective, efficient, and safe way, seeking to reduce the direct and indirect consequences of COVID-19.^6^ These transformations, however, are not an easy task.

The study by Ghisi et al.,^7^ published in the ABC Cardiol, despite having a small sample and using an online questionnaire developed by the authors, shows how the economic threats experienced by health professionals and their programs and the inability of many patients to have access and to be able to navigate in the virtual world greatly affected the participants of the EBCR. The authors assessed participants in EBCR programs on health literacy, technology use, and internet access and how they perceived their health during the pandemic, among other things, 26% reported worsening heart conditions and symptoms such as chest pain, shortness of breath, heart palpitations, anxiety, and depression. Going beyond also alerted to the fact that EBBR institutions and professionals should work to develop a better practical approach to monitor cardiac patients virtually and personalize preventive care, helping these individuals in their recovery and the prevention of events.^7^ Haskiah et al. also found a reduction in exercise capacity and an increase in fat percentage in participants who suspended their treatments during the COVID-19 pandemic. They suggest remote cardiac rehabilitation may be an effective alternative to outpatient cardiac rehabilitation in periods like the COVID-19 pandemic and even for patients who choose this approach.^8^ Although referral is a Class I recommendation, only 30-50% of eligible patients are referred for EBCR by their cardiologist, and even fewer manage to complete a program.^9,10^ Nakayama et al. They identified in Japan what also occurs in Brazil: the main impediment to attending EBCR programs is the distance from home to the program location and the scarcity of structured services.^10,11^

A positive factor in this new scenario of distance-supervised EBBR is the implementation of the so-called phase 4 of rehabilitation with exercises. This phase is characterized by the patient being no longer physically linked to the treatment centers but remotely. Ownbey et al.^12^ showed no significant differences in patient outcomes between hybrid and face-to-face care, indicating that remote EBCR is a viable addition to face-to-face care.^12^ However, in Brazil, as in other countries,^13^ most healthcare professionals have little or no experience implementing home and remote RCBE; in addition, medical insurance and the unified health system (SUS) usually do not cover this type of care. Greater investments are needed in training professionals and implementing remote exercise monitoring, education for healthy living, and behavior modification to improve overall health.

The EBCR is on a frontier, requiring further research in the area and greater investments that allow maintaining a greater number of patients in both face-to-face and remote care, where the greatest impediment is found in financial investment, patient education for self-monitoring, thus allowing greater safety concerning the prescription of physical exercise at a distance.
